# Merging of two level-1 trauma centers in Amsterdam: premerger demand in integrated acute trauma care

**DOI:** 10.1007/s00068-023-02287-9

**Published:** 2023-06-08

**Authors:** Eva Berkeveld, Wietse P. Zuidema, Kaoutar Azijli, Marleen H. van den Berg, Georgios F. Giannakopoulos, Frank W. Bloemers, Trauma Surgery research collaborative group, Veerle Cuijpers, Anissa Mahraoui, Jesse Moorees, Gulsum Z. Nasim

**Affiliations:** 1 Department of Trauma Surgery, Amsterdam UMC location Vrije Universiteit Amsterdam, De Boelelaan 1117, 1081 HV Amsterdam, The Netherlands; 2 Department of Emergency Medicine, Amsterdam UMC location Vrije Universiteit Amsterdam, De Boelelaan 1117, Amsterdam, The Netherlands; 3 Dutch Network for Acute Care North West, Amsterdam, The Netherlands; 4 Department of Trauma Surgery, Amsterdam UMC location University of Amsterdam, Meibergdreef 9, Amsterdam, The Netherlands

**Keywords:** Merger, Concentration, Trauma care, Hospital, Trauma center centralization, Polytrauma

## Abstract

**Purpose:**

Availability of adequate and appropriate trauma care is essential. A merger of two Dutch academic level-1 trauma centers is upcoming. However, in the literature, volume effects after a merger are inconclusive. This study aimed to examine the premerger demand for level-1 trauma care on integrated acute trauma care and evaluate the expected demand on the system.

**Methods:**

A retrospective observational study was conducted between 1-1-2018 and 1-1-2019 in two level-1 trauma centers in the Amsterdam region using data derived from the local trauma registries and electronic patient records. All trauma patients presented at both centers’ Emergency Departments (ED) were included. Patient- and injury characteristics and data concerning all prehospital and in-hospital-delivered trauma care were collected and compared. Pragmatically, the demand for trauma care in the post-merger setting was considered a sum of care demand for both centers.

**Results:**

In total, 8277 trauma patients were presented at both EDs, 4996 (60.4%) at location A and 3281 (39.6%) at location B. Overall, 462 patients were considered severely injured patients (Injury Severity Score ≥ 16). In total, 702 emergency surgeries (< 24 h) were performed, and 442 patients were admitted to the ICU. The sum care demand of both centers resulted in a 167.4% increase in trauma patients and a 151.1% increase in severely injured patients. Moreover, on 96 occasions annually, two or more patients within the same hour would require advanced trauma resuscitation by a specialized team or emergency surgery.

**Conclusion:**

A merger of two Dutch level-1 trauma centers would, in this scenario, result in a more than 150% increase in the post-merger setting’s demand for integrated acute trauma care.

## Introduction

Worldwide, inclusive centralized trauma systems have shown to be beneficial for patients’ survival, warranting the appropriate care at the right place [1, 2]. In the Netherlands, a mature, inclusive trauma system is adhered to, composed of a collaborative trauma system among eleven trauma regions. Due to the structure of level-1, -2, and -3 trauma centers, trauma care on various levels of complexity can be provided within each region [3].

High-complexity trauma care is provided in level-1 trauma centers, as these centers are equipped with the required expertise and resources to provide care for the severely injured (Injury Severity Score (ISS) ≥ 16). Additionally, patients with physiologic or neurologic instability with, if necessary, requirements for emergent neurosurgical or cardiothoracic interventions and patients sustaining a complex isolated injury can receive adequate treatment accordingly [3]. The literature showed that for severely injured patients, direct transportation to a level-1 trauma center decreases morbidity and mortality rates [4–6]. To support this, novel Dutch standards aim for > 90% of severely injured patients to be directly transported to a level-1 trauma center [7–9]. Due to the expertise role that level-1 trauma centers fulfill, ensuring the continuous availability of level-1 trauma care is essential.

Currently, in the Amsterdam area, for the first time in the Netherlands, a merger of two academic level-1 trauma centers is upcoming. Due to the consequent change in the catchment area in case of mergers [10], changes in patient input could be expected. Therefore, assessing and early intercepting potential capacity barriers along the integrated acute trauma care system seems imperative to warrant the continuity of trauma care in the post-merger setting.

However, anticipating the expected capacity demand in the post-merger setting is challenging, as, in the literature, effects on volume after a merger are inconclusive [11–14]. Some studies found a reduction in demand for capacities, such as reduced activity or total staffing, after analyzing large cohorts with different hospital sizes and merger types [11, 12]. In contrast, disappearing effects in operating efficiency were found after adjusting for the control group by Alexander et al. in a large sample of mergers [13]. The variety of volume effects after a merger is further underlined in a report by The Netherlands Authority for Consumers and Markets (ACM), demonstrating a range from a fall of 12% to a volume rise of more than 25% concerning twelve general hospital mergers in the Netherlands [14].

Moreover, due to the novel situation of merging two academic level-1 trauma centers in a mature, inclusive trauma system, extrapolating the volume effects of previous mergers to our upcoming post-merger setting is challenging. Therefore, this study aimed to examine the current demand for level-1 trauma care in integrated acute trauma care and evaluate the expected demand on the system by providing a comprehensive baseline situation in the premerger setting.

## Methods

### Study setting

The level-1 trauma centers Amsterdam UMC location VUmc (location A) and Amsterdam UMC location AMC (location B) are situated in Amsterdam and provide level-1 trauma care for two trauma regions, i.e., the provinces of North Holland and Flevoland [15]. Together, these regions cover an area of more than 3.5 million inhabitants. Both trauma centers are equipped with two modern trauma resuscitation rooms to resuscitate severely injured patients with (suspected) respiratory-, physiologic- or neurological abnormalities. A two-tiered trauma team activation is adhered. Based on a standardized triage protocol either a complete or selective trauma team is activated to treat the patient in the trauma resuscitation room [16]. During day time, a complete team consists of a trauma surgeon, emergency physician, two ED nurses, anesthesiologist, nurse anesthetist, radiologist, two diagnostic radiographers, intensivist, and neurologist. During evening and night shifts, a surgical registrar and resident are present at the start of the resuscitation and the on-call trauma surgeon is present within 15 min. In contrast, a selective trauma team consists of an emergency physician, emergency resident, ED nurse, radiology resident, and diagnostic radiographer [8] Specially set up Emergency Departments (ED), Operating rooms (OR), and Intensive Care Units (ICU) function to provide adequate level-1 trauma care. Together, these essential components form a streamlined, collaborative integration to provide patients with definitive treatment as swiftly as possible. In the prehospital setting, care is provided by highly trained and experienced Emergency Medical Services (EMS) crews additionally supported by a physician-staffed Helicopter Emergency Medical Services (HEMS) crew to provide A(T)LS care [17]. Both level-1 centers feature a helicopter landing platform, whereas the permanent pitch of the HEMS Lifeliner-1 is situated at location A.

The merger of the two level-1 trauma centers was enacted as part of the merger of two academic hospitals in Amsterdam. The latter aims to ensure the most qualitative care for patients with complex and rare diseases and high-quality emergency care on a 24–7 basis. Additionally, it provides an opportunity to expand scientific knowledge, and educational practices benefit from shared expertise [18]. Concerning trauma care, the merger supports meeting the required 240 severely injured to be treated at a level-1 trauma center annually, required to warrant quality and staff efficiency. Following the decision to merge the two academic hospitals, locations A and B, it was decided in the interest of the entire healthcare system to concentrate all acute care at location B. Consequently, for this reason, the two level-1 trauma centers were planned to merge into location B.

### Study design

This retrospective study analyzed all data of two academic level-1 trauma centers in Amsterdam between January 1st, 2018, and January 1st, 2019. All trauma patients presented at the trauma centers’ Emergency Departments (ED) were included. Patient characteristics, prehospital, in-hospital, and outpatient clinic information were derived from the Dutch National Trauma Registry and complemented with Electronic Patient Record information. Prehospital characteristics included the location of the scene, method of transportation, transport time, and HEMS assistance. In-hospital information was composed of arrival date and time, length of stay (LOS) at ED, OR, ICU, and clinical departments. In addition, resuscitation-specific information was collected, such as trauma resuscitation team activation at ED, patient’s ISS, and CT-scan usage at ED. ISS from non-admitted patients was considered as an ISS below 16.

If a patient required an intervention, information regarding the urgency of the intervention (performed within 24 h after arrival) and the number of interventions performed were collected. Additionally, admissions of ICU and clinical (trauma surgical, pediatric or neurologic department where the trauma surgeon provides (co-)treatment were collected. In-hospital mortality, destination after discharge, and the number of outpatient clinic visits were retrieved.

### Data analysis

Descriptive statistics were used. Categorical variables were presented as percentages, whereas continuous variables were presented as mean (standard deviation (SD)) or median with interquartile range (25th–75th percentiles). Data were analyzed using IBM SPSS Statistics version 24.0 (IBM, New York, USA). Prehospital time distances were calculated using a route planner accounting for one day of the week and the time of day. Data from level-1 trauma center locations A and B were added to provide an overview of the sum of both capacities in case of concentration at location B in the post-merger setting. Patient input per shift (i.e., day 8.00 AM till 4.00 PM, evening 4.00 PM till 11.00 PM, and night 11.00 PM till 8.00 AM) per 24 h and annum were examined.

## Results

### Descriptives

In total, 8277 trauma patients were presented at both EDs during the study period, 4996 (60.4%) at location A, and 3281 (39.6%) at location B (Table 1). Excluded were duplicates, patients not presented at the ED, and patients left on their behalf before diagnostics commenced. The total study population predominantly consisted of male patients (59.6%) with a mean age of 40.9 (SD 25.0), as shown in Table 1. Overall, 462 patients were considered severely injured (ISS ≥ 16), of which 278 (60.2%) were presented at location A and 184 (39.8%) at location B. In total, 1799 patients were resuscitated at the trauma resuscitation room by a trauma team, including all severely injured patients (*n* = 462). The mean ISS of the admitted patients (*n* = 2509) was 9.3 (SD 9.2).

**Table 1:** Demographic characteristics

	Location A (*n* = 4996)	Location B (*n* = 3281)	Total (*n* = 8277)
Age, mean (SD)	41.6 (25.0)	39.8 (23.9)	40.9 (25.0)
Gender, male (%)	2911 (55.3)	2025 (61.7)	4936 (59.6)
ISS, mean (SD) *admitted patients only*	9.7 (9.7)	8.7 (8.5)	9.3 (9.2)
Destination after discharge, *n* (%)			
Home	4417 (88.4)	2915 (88.8)	7332 (88.6)
Nursing home	162 (3.2)	30 (0.9)	192 (2.3)
Physical rehabilitation center	54 (1.1)	33 (1.0)	87 (1.1)
Other hospital	270 (5.4)	230 (7.0)	500 (6.0)
Left against medical advice	19 (0.4)	19 (0.6)	38 (0.5)
Deceased in-hospital	74 (1.5)	54 (1.7)	128 (1.5)

*ISS* Injury Severity Score

### Integrated acute trauma care

**Fig. 1:**
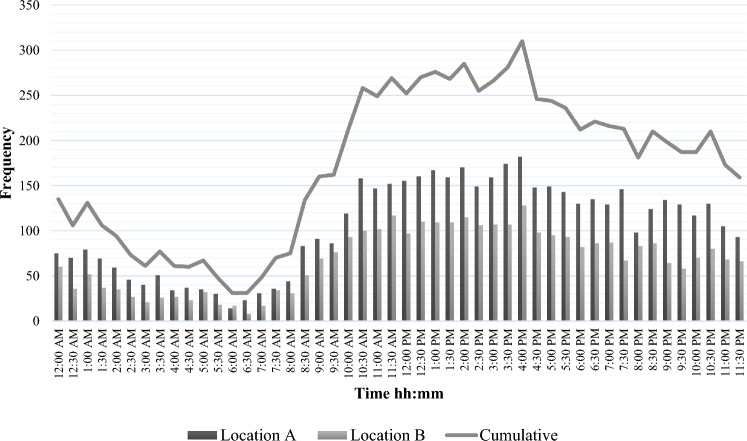
Timing of trauma patients’ arrival at ED (*n* = 8277). *ED* Emergency Department

**Fig. 2:**
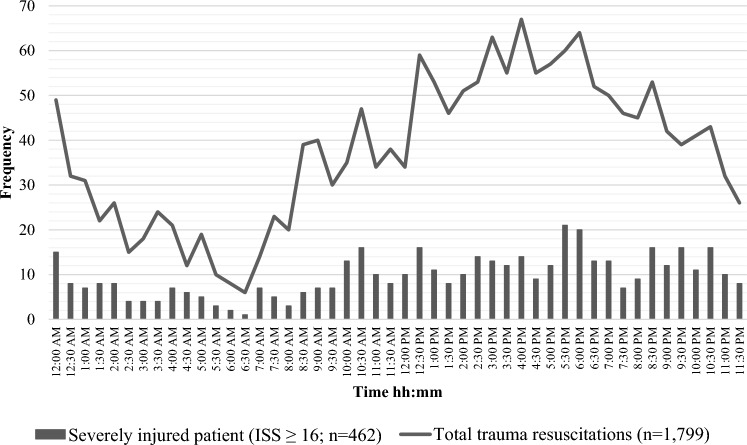
Timing of patients’ arrival resuscitated by a specialized trauma team at the trauma resuscitation room in ED. *ED* Emergency Department, *ISS* Injury Severity Score

**Fig. 3:**
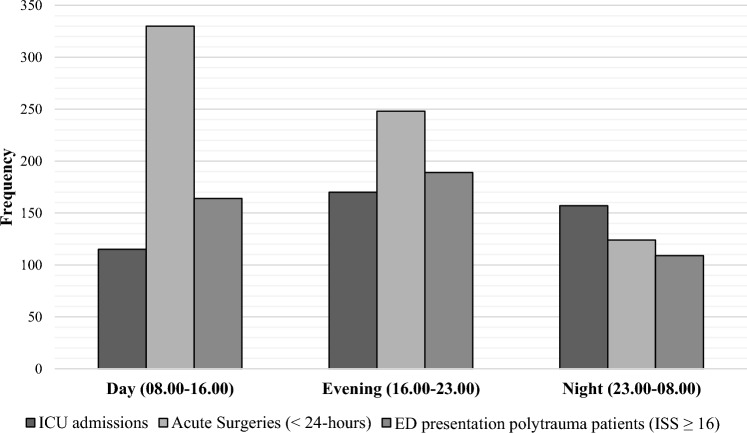
Trauma care necessity per shift in both trauma centers. *ED* Emergency Department, *ICU* Intensive Care Unit, *ISS* Injury Severity Score

Table 2 shows that for the total study population, the vast majority of patients arrived at ED directly from the accident scene (95.7%), and the most common method of transportation was by EMS (55.1%). For both trauma centers, patients’ arrival time at ED shows highest during day and evening shifts (Fig. 1). Similarly, advanced trauma resuscitations by a specialized team, including for severely injured patients, occurred most frequently during the day and evening shifts (Fig. 2). Most emergency interventions were performed during day and evening shifts and were required for 702 patients in total (Table 3), whereas the necessity for ICU admission (*n* = 442) was most prevalent during the evening (38.5%) and night (35.5%) shifts (Fig. 3).

**Table 2:** Prehospital characteristics

	Location A (*n* = 4996)	Location B (*n* = 3281)	Total (*n* = 8277)
Transported from, *n* (%)			
Accident scene	4837 (96.8)	3080 (93.9)	7918 (95.7)
Hospital referral	51 (1.0)	76 (2.3)	127 (1.5)
General practitioner referral	43 (0.9)	20 (0.6)	63 (0.8)
Missing	64 (1.3)	105 (3.2)	169 (2.0)
Method of transportation, *n* (%)			
EMS	2758 (55.2)	1801 (54.9)	4559 (55.1)
Own transportation	905 (18.1)	1449 (44.2)	2354 (28.5)
HEMS	12 (0.2)	–	12 (0.1)
Other	1321 (26.5)	31 (0.9)	1352 (16.3)
HEMS assistance, *n* (%)	251 (5.0)	86 (2.6)	337 (4.1)

*EMS* Emergency Medical Services, *HEMS* physician staffed Helicopter Emergency Medical Services

**Table 3:** In-hospital characteristics

	Location A (*n* = 4996)	Location B (*n* = 3281)	Total (*n* = 8277)
Emergency Department			
Trauma resuscitation room presentation, *n* (%)	941 (18.8)	858 (26.2)	1799 (21.7)
General ED room presentation, *n* (%)	4055 (81.2)	2423 (73.8)	6478 (78.3)
CT-scan, *n* (%)	1823 (36.5)	1226 (37.4)	3049 (36.8)
LOS ED with trauma resuscitation (hh:mm), median (IQR)	3:55 (2:50)	2:54 (2:30)	
LOS ED general (hh:mm), median IQR	2:44 (2:37)	2:33 (2:19)	
Operating room			
Emergency surgery (< 24 h), *n* (%)	392 (7.8)	310 (9.4)	702 (8.5)
Clinical departments			
Admission, *n* (%)			
ICU	237 (4.7)	205 (6.2)	442 (5.3)
Clinic	1209 (24.2)	847 (25.8)	2056 (24.8)
LOS, median (IQR)			
ICU	3.0 (5.0)	2.0 (3.0)	
Clinic	4.0 (7.0)	3.0 (5.0)	

*ED* Emergency Department, *LOS* length of stay, *ICU* Intensive Care Unit

### Sum of trauma care

**Fig. 4:**
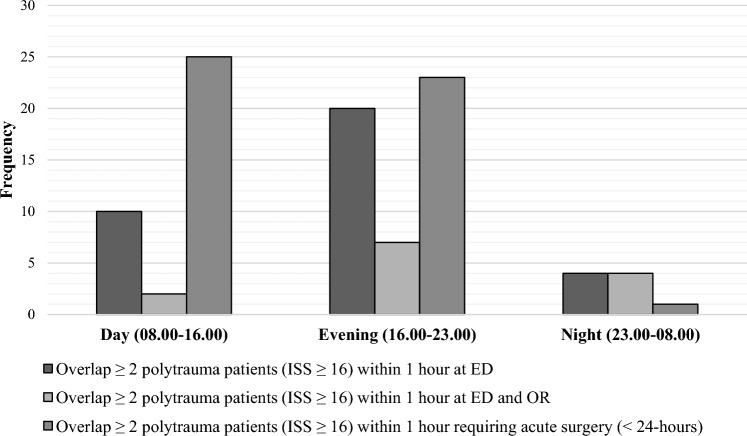
Severely injured patients’ (Injury Severity Score ≥ 16) overlap in both trauma centers. *ED* Emergency Department, *OR* Operating Room, *ISS* Injury Severity Score

Adjustment for trauma care from both centers to be solely concentrated at location B resulted in a 167.4% increase in trauma patients and a 151.1% increase in severely injured patients. Median transport time from the scene to location B increased by 5.4 min (IQR 4.0) from 12 to 17 min (*n* = 1029). The number of advanced trauma resuscitations (including for severely injured patients) at the ED increased by 109.7%, from 858 to 1799. This increase would be 150.0%, 160.9%, and 153.5%, respectively, during the day-, evening- and night shifts. For severely injured patients specifically (*n* = 462), an increase of 124.7% during the day shifts, 170.0% during the evening shifts, and 165.9% during the night shifts was found. In total, on 34 occasions arrival of two or more consecutive patients would occur within one hour, requiring advanced trauma resuscitation by a specialized trauma team in ED (Fig. 4). During the evening and nighttime shifts, two or more patients within one hour requiring either advanced trauma resuscitation at ED or emergency surgery would occur on 59 occasions annually.

## Discussion

This study examined the current premerger demand for level-1 trauma care in integrated acute care involving two level-1 trauma centers in the Amsterdam region. During the study period, 8277 patients required trauma care, of which 60.4% at location A and 39.6% at location B. Overall, 462 patients were considered severely injured. Due to the expert care delivered in level-1 trauma centers, it is crucial to ensure the appropriate availability of capacity of staff and resources. Based on varying volume effects after merger described in the literature [11–14], the extrapolation of an expectation model to the current situation is challenging. Pragmatically, the demand for trauma care in the post-merger setting considered as a sum of care demand of both centers resulted in a 167.4% increase in trauma patients and a 151.1% increase in severely injured patients for location B. Moreover, on 96 occasions annually, two or more patients within the same hour would require advanced trauma resuscitation by a specialized team or emergency surgery.

To manage increased patient input on a tactical and operational level, the literature emphasizes the importance of optimizing throughput and output components of acute care flow [19]. Early assessment of potentially required adaptations in the post-merger center seems preferable. Mentzoni et al. detected an increased input of 40.9% and a corresponding rise in LOS of 20.9% the first year after their Norwegian catchment area was reconfigured by 44%. This increased input expanded further during peak hours [20]. To prevent crowding and a prolonged LOS in ED, several capacities-enhancing strategies include optimizing triage, increasing the number of staffed beds, and installing additional wards as temporary in-patient dispositions [3, 19–22].

Generally, reducing LOS in academic hospitals is challenging due to the complexity of care and treatments [23, 24]. Together with the admission rate, it forms a substantially important factor influencing hospital-level flow through a unit [25], and is highly dependent on the urgency of admission and admission season [26, 27]. This current study found a median LOS in ED of 2:44 h for location A and 2:33 h for location B, which is similar to the national ED LOS in our inclusive trauma system [28] and mirrors previous findings from a different Dutch level-1 trauma center [29]. For patients requiring advanced care at the trauma resuscitation room, mean times of 3:55 h (location A) and 2:54 h (location B) were found. This duration included the time spent in a regular ED bed after their trauma resuscitation was completed when no further care at the trauma resuscitation room was required. Furthermore, It was shown by McCarthy et al. that decreasing the number of patients waiting to ‘board’ to their in-patient disposition has the greatest benefits for flow efficiency and overcoming ED crowding [21]. Therefore, considering the two merging academic level-1 trauma centers, one strategy to reduce LOS could be increasing the number of staffed beds in the clinical trauma wards and temporary disposition wards [30]. This would contribute to ED output by decreasing ED boarding time and aid the overall increase in patient input over all three shifts ranging from 150.0 and 160.9%, as depicted in this study’s results.

In addition, due to the emergent character of trauma care, a specialized team can be ‘fixed and saturated’ with resuscitating one severely injured patient. This study found that the arrival of severely injured patients in the ED would mainly occur during the evening and night shifts. The overlap of two patients requiring advanced trauma resuscitation in ED within one hour would be most common during evening shifts. Therefore, attention to providing adequate staffing and resources for initial trauma resuscitation in ED during these shifts could contribute to optimizing patient throughput. To achieve this in the post-merger setting, installment of 24–7 in-house trauma surgeon presence and stand-by coverage by an additional trauma surgeon can aid in warranting continuity of care.

Moreover, besides, after severe trauma, a patient often requires further resuscitative management, such as damage control surgery or primary fracture care in an emergency setting and further definitive care at the ICU. Consequently, OR and ICU admission availability is essential to warrant level-1 trauma care delivery. In total, the amount of ICU admissions accounted for 17.7% of all trauma-related admissions. Adding up both centers would result in a 115.6% increase in trauma-related ICU admissions, mainly during the evening and night shifts. Therefore, to comply with the current standards from the Dutch Trauma Society (NVT) for a level-1 trauma center to always have one ICU bed available at all times to admit a severely injured patient [3], expanding the minimum availability to two ICU beds might be required to cope with the potential increase in the post-merger setting. Capacity-enhancing strategies include facilitation for ICU patients who require observation due to, for example, costal fractures to be monitored at the Post Anesthesia Care Unit (PACU). Also, to preserve the continuity of the OR schedule, the intensivist will aim to replace the anesthesiologist’s role during trauma resuscitation activations in ED. This way, the (emergency) OR will be minimally impacted regarding staffing availability.

Concerning staff expansion, it is advantageous that the attending intensivists already work in a shared staffing pool between the two centers. However, high demand exists for (specialized) nurses in the trauma resuscitation room, OR, ICU, and clinical ward [31]. Therefore, concerning capacity expansion, the most significant bottleneck will likely be facilitating adequate nursing staffing. Overall, investment in staffed beds in the clinical trauma ward benefits a two-tiered strategy, as it supports both output flow in ED and ICU [32]. Naturally, optimal flow towards the clinical trauma ward comes hand in hand with ensuring adequate availability by preventing a stagnating output flow in the clinical department. The majority of patients in the clinical ward were discharged to their own living environment (74.7%), with additional home care if necessary, whereas 9.6% of patients were discharged to a nursing home and 4.6% to a rehabilitation clinic. The corresponding arrangements and transfer to continue the required care at home, nursing home, or rehabilitation clinic usually only occur during weekdays. In the case of admitted clinical patients for whom hospital care is no longer necessary, delays concerning organizational and logistical aspects of discharge disposition cause unnecessary hospital bed occupancy. While the number of nursing- or rehab capacity would not change in the catchment area due to the merger, arranging the patients to be dispositioned to these beds might be more challenging. The literature showed that multidisciplinary attention to discharge planning has effectively reduced unnecessary LOS [33]. An additional improvement to overcome this could include optimizing discharge possibilities on a 7-day per week basis and continuing the required care in the (nursing-)home or rehabilitation setting.

Altogether, the in-hospital care flow forms a connected entity dependent on individual departments’ flow and collective collaboration. Besides the anticipated volume effects caused directly by the merger, due to the integral character of trauma care, regional restructuring of patient flow seems necessary to meet the novel > 90% standards for severely injured patients in the post-merger setting [7]. Several studies showed undertriage rates between 21.6 and 34.6% among various Dutch trauma regions [34–37]. To aim for severely injured patients to be directly transported to the level-1 trauma center, triage should be enhanced for the ‘potentially severely injured patients’ to be evaluated in the level-1 trauma center and thus reduce undertriage. A clear two-way interaction with level-2 trauma centers should be established to mitigate this increased patient input. That way, patients who, after evaluation in the level-1 trauma center, do not sustain severe injury and otherwise do not benefit from level-1 trauma care can be transferred safely to a surrounding level-2 trauma center when necessary.

In this study, the retrospective inclusion of all trauma patients presented through the ED with acute traumatic injury might have caused to some extent, selection bias. Patients admitted via other (e.g., elective) routes might have been missed. However, focusing on the patients admitted via ED provided a thorough insight into the patient’s integrated care flow. Patient flow is rather erratic and non-linear [38]. In addition, the analysis of the transport time difference in the post-merger setting only included admitted patients from location A from whom the scene location was available. The present study was conducted assuming that a merger would directly translate into a sum of the demand for care from both trauma centers. Despite it being uncertain whether a merger results in this input, in the literature, effects on volume after a merger are controversial [14] and do not readily generalize to the trauma system setting. Nevertheless, generally, in acute care, one large unit is more efficient than two small ones [32], which is in line with one of the general strategies of hospital mergers to reduce duplication of services [10, 12]. Therefore, the sum of capacity adhered in this current study might function as a maximum baseline for the required capacity.

## Conclusion

This study examined the current premerger demand for level-1 trauma care involving two level-1 trauma centers in the Amsterdam region. Based on premerger data from 2018, a sum of capacity demand would result in a more than 150% increase in the post-merger setting’s integrated acute trauma care, including for the severely injured. These data are essential for successfully integrating two major trauma centers in Amsterdam. Future research is recommended to evaluate volume effects in the post-merger setting.

## Data Availability

Original data remain available and access may be provided upon reasonable request.
